# Nostrums and the Clergy

**Published:** 1881-01

**Authors:** 


NOSTRUMS AND THE CLERGY.—It is astonishing how many clerical names are to be found appended to, and endorsing the numerous quack nostrums of the present day! These gentlemen evidently do not take the trouble to think properly about the matter ere the certificates bearing their names are signed, for if they did, certainly very few, if any endorsations of such preparations would be made. In the first place it is simply absurd to suppose that any remedy, however valuable, or combination of remedies, is capable of curing several diseases, much less all varieties of disease, as is generally claimed in the advertisements of secret nostrums! But it has been found upon analysis that many of the preparations so highly lauded and endorsed, in this way are inert, or else of incompatible substances. The endorsation of such vile or valuless preparations, by the clergy, induces their purchase by persons who are ignorant of what they are; and thus fraud and injury to health are often the result. Physicians know also, that even life is sometimes destroyed by their use, simply because they had the recommendation of some clergyman of the victim's acquaintance. When physicians, who know the character of these vile, or worthless preparations, inveigh against thier use, they are often told that the Rev. Mr., or Rev. Doctor so and so, says the remedy is a good and safe one, and surely he would not recommend anything that was not right and proper to be used. He is too good a man to endorse a valueless or dangerous compound!

But if the knowledge of theology possessed by these gentlemen should be measured by their acqaintance with the therapeutic properties, or often want of valuable properties, at all, of the nostrums they so strongly endorse, they must be considered by intelligent physicians at least, as blind leaders of the blind, on the way to Paradise.

The late Dr. Thomas E. Bond of this city, became a minister of the Methodist Church, and for several years ably edited a religious newspaper here; but he established a rule to exclude patent medicines, and nostrums, from the *advertising department* of *his* paper entirely. He did this too, in several cases, at large pecuniary loss to his enterprise. But such was his determination to keep his hands and conscience clear in the premises, that he permitted no price to change his purpose of keeping *all secret nostrums* from the columns of his paper. This stand against quackery was taken several years after he had given up the practice of his profession. Here was an example that *all* clergymen would do well to follow. He not only declined to countenance secret nostrums in every way, but manfully refused to allow the endorsements of them by others to appear for pay, in the advertising columns of *his* religious publication. There are several strong lights in which this nefarious business, for extorting money from a credulous public, can be seen, and in most of them the endorsing clergy occupy an unenviable position, but we must close our remarks for the present.

